# Enzymatic Synthesis of an Undecorated Capsular Polysaccharide from *Campylobacter jejuni*

**DOI:** 10.1021/acs.biochem.5c00314

**Published:** 2025-08-11

**Authors:** Dao Feng Xiang, Tamari Narindoshvili, Frank M. Raushel

**Affiliations:** Department of Chemistry, 14736 Texas A&M University, College Station, Texas 77843, United States

## Abstract

The exterior surface of the human pathogen *Campylobacter jejuni* is coated with a capsular polysaccharide (CPS) that helps protect it from the host immune system. In *C. jejuni* NCTC 11168 the repeating linear polysaccharide is composed of d-ribose, *N*-acetyl-d-galactosamine and d-glucuronic acid that is further amidated with either ethanolamine or serinol. The CPS is also decorated with d-*glycero*-l-*gluco*-heptose and methyl phosphoramidate. We have now shown that the polymerization of the undecorated CPS requires the sequential activity of six unique enzymes that must act in concert with one another. The catalytic activity of these six enzymes enabled a robust synthetic strategy to be developed to facilitate the assembly and isolation of specific oligosaccharides of up to 10 units in length. Modifications to this strategy enabled the isolation of mixtures containing oligosaccharides containing at least 19 monomeric units. The oligosaccharides were isolated by anion exchange chromatography and chemically characterized using ESI mass spectrometry and ^1^H NMR spectroscopy. These oligosaccharides will enable the reaction mechanisms for the decoration of the capsular polysaccharides to be determined and may facilitate the development of glycoconjugate vaccines.

## Graphical abstract

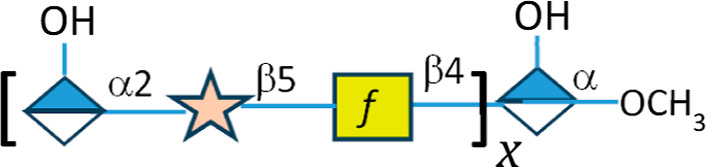

## Introduction

The leading cause of food poisoning in North America and Europe is the Gram-negative bacterium *Campylobacter jejuni*.^1–3^ The exterior surface of this pathogenic bacterium is coated with a capsular polysaccharide (CPS) that provides structural stability and maintenance of the bacterial cell wall.^4,5^ The CPS also helps to protect the organism from the host immune system.^6^ The capsular polysaccharides of the various strains and serotypes of *C. jejuni* have different repeating sequences of modified and unusual sugars.^7^ To date more than 35 unique serotypes have been identified and at least 12 capsular polysaccharides have been chemically characterized.^7,8^ The CPS is apparently attached to a poly-KDO (3-deoxy-d-manno-oct-2-ulosonic acid) linker that is in turn attached to a diacyl glycerophosphate anchor to the outer membrane.^9,10^ There are currently no FDA-approved vaccines for the prevention of *C. jejuni* infections, but the best candidates appear to be glycoconjugates that mimic the structures of the capsular polysaccharides.

The repeating structural unit in the CPS from *C. jejuni* NCTC 11168 is shown in Figure 1 and consists of d-ribose (Rib), *N*-acetyl-d-galactosamine (Gal*f*NAc) and d-glucuronic acid (GlcA).^7,11^ The GlcA moiety is further modified by amidation with either serinol (compound **1**) or ethanolamine (compound **2**). An additional modification of the GlcA moiety includes glycosylation with d-*glycero*-l-*gluco*-heptose at C3, which is further methylated at C6 and methyl phosphoramidation at C4. An additional methyl phosphoramidation is found at C3 of the Gal*f*NAc moiety.

**Figure 1:**
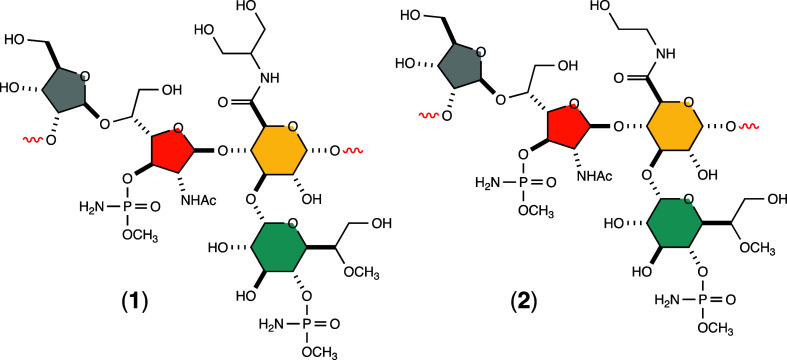
Two repeating structural units in the capsular polysaccharide of *C. jejuni* NCTC 11168 (serotype HS:2). The repeating structural unit consists of d-ribose (highlighted in gray), *N*-acetyl-d-galactosamine (highlighted in orange), d-glucuronic acid (highlighted in yellow) and d-*glycero*-l-*gluco*-heptose (highlighted in blue). In structure **1** the glucuronic acid is amidated with serinol, whereas in structure **2** the amide is made from ethanolamine.

The gene cluster that encodes for most, but not all, of the enzymes required for the biosynthesis of the CPS of *C. jejuni* NCTC 11168 is presented in Figure 2.^12^ Those genes highlighted in green are required for the synthesis and transfer of the methyl phosphoramidate group to the ribose and heptose moieties.^13–16^ The genes required for the biosynthesis and transfer of the heptose moiety are highlighted in blue.^17–23^ Cj1441 catalyzes the oxidation of UDP-glucose to UDP-glucuronic acid, Cj1437 catalyzes the deamination of dihydroxyacetone to form serinol-phosphate, and Cj1436 catalyzes the decarboxylation of serine-phosphate to ethanolamine phosphate.^24,26^ The yellow-colored genes in Figure 2 are of unknown function.

**Figure 2:**
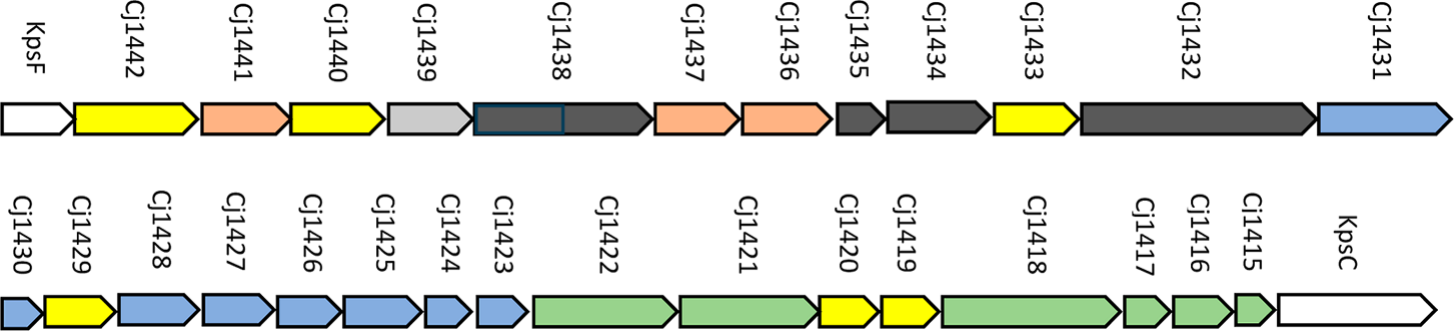
Gene cluster for the biosynthesis of the capsular polysaccharide in *C. jejuni* NCTC 11168 (serotype HS:2). The genes colored green are associated with the synthesis and transfer of the methyl phosphoramidate group to the polysaccharide chain. The genes colored blue are required for the synthesis and transfer of the d-*glycero*-l-*gluco*-heptose moiety to the capsular polysaccharide. The proteins encoded by the genes colored yellow are of unknown function. The salmon-colored gene are required for the biosynthesis of d-glucuronate, serinol-phosphate, and ethanolamine-phosphate. The gray colored gene is required for the conversion of UDP-NAc-d-galactopyranose to UDP-NAc-d-galactofuranose. The genes colored black are required for the polymerization of the CPS. Additional details are provided in the text.

We have recently elucidated the six enzymatic reactions that are required to polymerize the repeating trisaccharide unit within the CPS of the HS:2 serotype of *C. jejuni*.^27–29^ During these transformations the C-terminal domain of Cj1432 (Cj1432_C_) catalyzes the transfer of ribose-5-phosphate from phosphoribosyl phosphate (PRPP) to C5 of Gal*f*NAc at the nonreducing end of the growing CPS.^29^ In the next step the middle domain of Cj1432 (Cj1432_M_) catalyzes the hydrolysis of phosphate from the hydroxyl group at C5.^29^ The N-terminal domain of Cj1432 (Cj1432_N_) then catalyzes the transfer of GlcA from UDP-GlcA to the hydroxyl group at C2 of the ribose moiety.^27^ The carboxylate of the glucuronic acid is then amidated with either serinol phosphate or ethanolamine phosphate (as shown in Figure 1) using the C-terminal domain of Cj1438 (Cj1438_C_), and then in the penultimate step in the cycle, the phosphate ester is hydrolyzed by Cj1435.^27,30^ Finally, either the N-terminal domain of Cj1438 (Cj1438_N_) or Cj1434 catalyzes the transfer of Gal*f*NAc from UDP-Gal*f*NAc to C4 of the glucuronamide moiety.^28^ These six steps are then repeated for the polymerization of the CPS that contains d-ribose, d-Gal*f*NAc, and the amide of d-GlcA. The specific enzymatic transformations are highlighted in Figure 3. Support for this reaction pathway has been obtained by the construction, isolation, and chemical characterization of the six trisaccharide motifs depicted in Figure 3 using a specific methyl glycoside as an initiating primer.^27–29^

**Figure 3:**
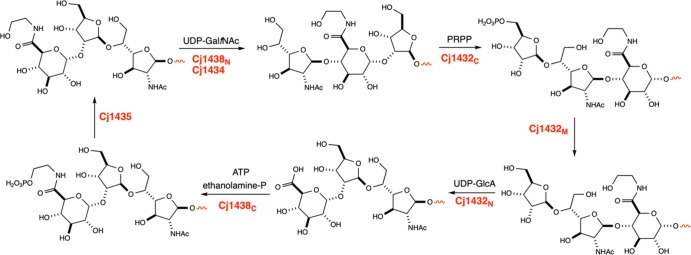
Reaction scheme for the polymerization of d-ribose, d-Gal*f*NAc, and d-GlcA during the biosynthesis of the CPS of *C. jejuni*, serotype HS:2. In this scheme we have shown the reaction catalyzed at the nonreducing end of the polymer by each enzyme in the cycle. Since three of these reactions (catalyzed by Cj1438_N_, Cj1438, Cj1432_C_, and Cj1432_N_) are making the polymer longer, we have arbitrarily removed one sugar moiety from the reducing end of polymer for those steps to make the figure manageable.

Here we have developed protocols for the enzymatic synthesis of specific oligomers of the HS:2 serotype that contain up to 19 monomeric units. These oligosaccharides may serve as the starting point for the generation of glycoconjugate vaccines and as novel substrates for the elucidation of the reaction mechanisms for the decoration of the backbone capsular polysaccharides with d-*glycero*-l-*gluco*-heptose and methyl phosphoramidate, as observed in the fully mature CPS from *C. jejuni* NCTC 11168.

## Materials and Methods

### Materials

Adenosine 5′-triphosphate (ATP), 5-phospho-d-ribose 1-diphosphate (PRPP), and ethanolamine phosphate were purchased from Sigma-Aldrich. UDP-d-glucuronic acid (UDP-GlcA) was purchased from Carbosynth. Growth medium lysogeny broth (LB) and isopropyl-β-d-thiogalactopyranoside (IPTG) were purchased from Research Products International. HisTrap columns, HiTrap Q HP anion exchange columns, and Vivaspin 20 (10 kDa MWCO) spin filters were obtained from Cytiva. The 10 K Nanosep spin filters were purchased from PALL Corporation (Port Washington, NY). The protease inhibitor cocktail (cOmplete Mini), DNase I, kanamycin, gentamicin, ampicillin, imidazole, and HEPES were purchased from Sigma-Aldrich. All other compounds, unless stated otherwise, were purchased from either Sigma-Aldrich or Thermo Fisher Scientific. UDP-*N*-acetyl-d-galactosamine furanose (UDP-Gal*f*NAc) and primer **3** (methyl glycoside of d-glucuronic acid amidated with ethanolamine, Figure 4) were chemically synthesized as previously reported.^28^ The structures of the substrates and products isolated for this investigation are provided in Figures 4 and 7. The chemical formulas, along with the measured and calculated *m*/*z* values for the various products, are listed in Tables 1 and 2.

**Figure 4:**
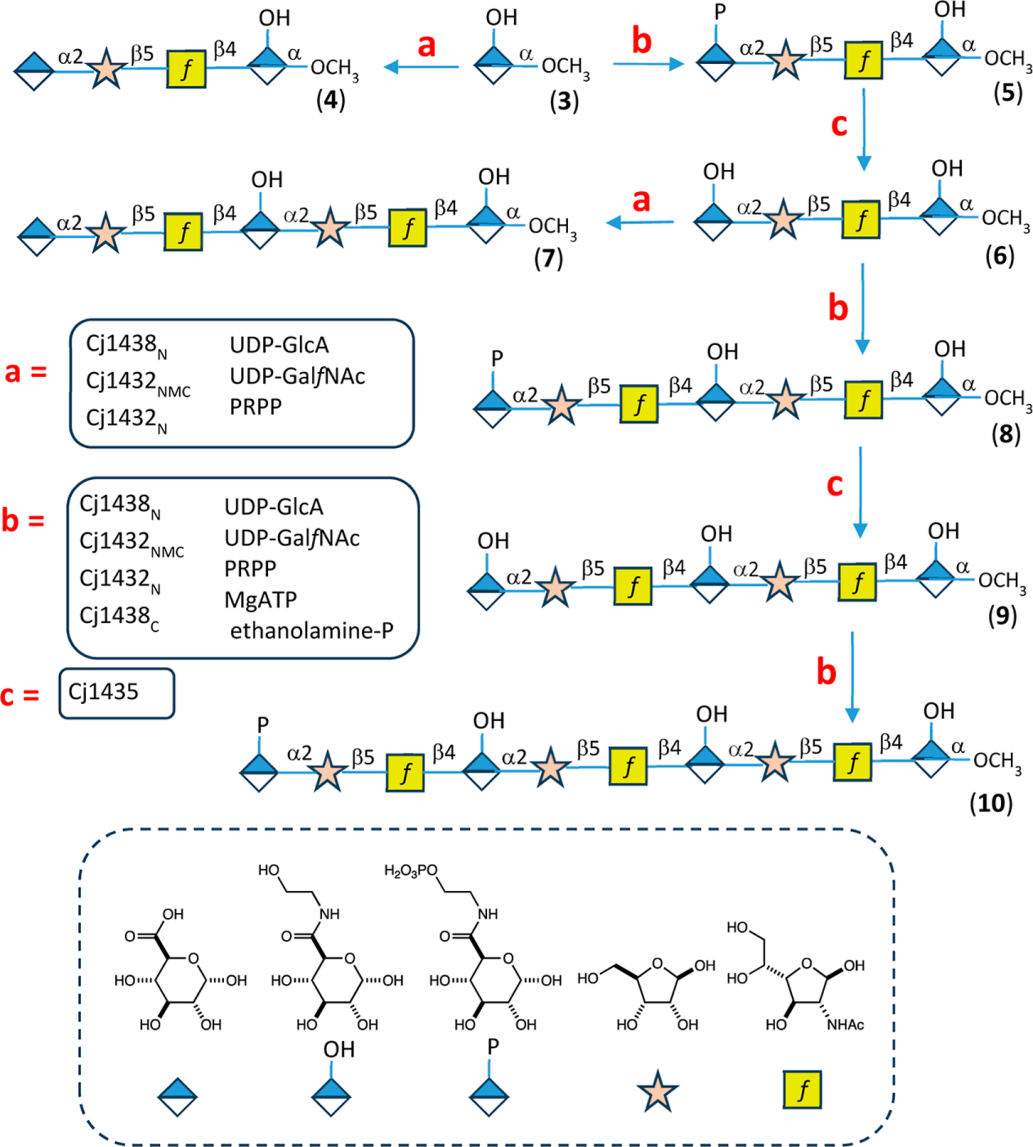
Outline for the enzymatic synthesis of defined oligosaccharides containing d-ribose, d-Gal*f*NAc, and the ethanolamine amide of d-GlcA. Additional details are found in the text.

**Table 1:** Mass Spectral Data for Enzyme-Synthesized Oligomers

oligomer	formula	theoretical mass [M – H^+^]^−^ *m/z* *z* = 1	relative abundance	measured mass [M – H^+^]^−^ *m/z* *z* = 1	relative abundance
tetramer **4**	C_30_H_52_N_3_O_25_P mol. wt. 885.72 amu	884.25	100.0	884.25	100.0
		885.26	32.4	885.26	37.1
		886.26	2.7	886.26	11.4
heptamer **8**	C_51_H_86_N_5_O_40_P mol. wt. 1440.22 amu	1438.45	100.0	1438.45	100.0
		1439.46	55.2	1439.46	62.0
		1440.46	14.9	1440.46	25.4
		1441.46	4.5	1441.46	9.1
decamer **10**	C_72_H_120_N_7_O_55_P mol. wt. 1994.72 amu	1992.65	100.0	1992.65	100.0
		1993.65	77.9	1993.65	85.6
		1994.65	11.3	1994.65	46.6
		1995.65	8.8	1995.65	19.4
		1996.66	3.4	1996.66	6.7

**Table 2:** Mass Spectral Data for Enzyme-Synthesized Oligomers

oligomer	formula	theoretical mass [M – 2H^+^]^2–^ *m/z* *z* = 2	relative abundance	measured mass [M – 2H^+^]^2–^ *m/z* *z* = 2	relative abundance
tridecamer **10–2**	C_93_H_154_N_9_O_70_P mol. wt. 2549.22 amu	1272.92	88.4	1272.92	94.0
		1273.42	100.0	1272.42	100.0
		1273.92	49.8	1273.92	70.8
		1274.42	14.4	1274.42	35.0
		1274.92	7.2	1274.92	14.8
		1275.42	4.8	1275.42	5.0
hexadecamer **10–3**	C_114_H_188_N_11_O_85_P Mol. wt. 3103.72 amu	1550.01	81.1	1550.01	73.0
		1550.51	100.0	1550.51	100.0
		1551.02	61.1	1551.02	82.9
		1551.52	17.5	1551.52	46.2
		1552.02	14.2	1552.02	17.2
nonodecamer **10–4**	C_135_H_222_N_13_O_100_P Mol. wt. 3656.24 amu	1827.11	68.5	1827.11	73.7
		1827.61	100.0	1827.61	85.7
		1828.11	72.0	1828.11	100
		1828.61	24.4	1828.61	52.4
		1829.11	10.7	1829.11	25.5
		1829.62	3.3	1829.62	11.4

### Equipment

The purification of proteins and products was conducted using an NGC Chromatography System (BIO-RAD). Ultraviolet spectra were collected on a SpectraMax ABS Plus UV–vis plate reader (Molecular Devices) using a 1 cm quartz cuvette. Nuclear magnetic resonance (NMR) spectra were recorded at 30 °C using standard pulse sequences on an Avance III 500 MHz NMR spectrometer equipped with a broad band probe and sample changer. Electrospray ionization mass spectrometry (ESI-MS) experiments were performed using a Thermo Scientific Q Exactive Focus instrument. Samples were injected into a 10 μL loop and transferred to the instrument using a mobile phase containing 70% methanol and 30% water with 0.1% formic acid at a flow rate of 600 μL/min. The Q Exactive Focus HESI source was operated in full MS in positive and negative modes. The mass resolution was tuned to 70,000 fwhm (full width at half-maximum) at *m*/*z* 200. The spray voltage was set to 3.5 kV for positive mode and 2.8 kV for negative mode. The vaporizer and transfer capillary temperatures were held at 250 and 320 °C, respectively. The S-Lens RF level was set at 50 v. Exactive Series 2.11/Xcalibur 4.2.47 software was used for data acquisition and processing.

### Plasmid Construction

The genes for Cj1432, Cj1438, and Cj1435 employed in this study are from *C. jejuni* NCTC 11168 (serotype HS:2). The full length gene for Cj1432 (minus the nucleotides for the C-terminal 117 amino acids; UniProt id: Q0P8I2; denoted here as Cj1432_NMC_), the N-terminal truncated gene for Cj1432 (1-356 amino acids; UniProt id: Q0P8I2; denoted here as Cj1432_N_), the N-terminal truncated gene for Cj1438 (1-325 amino acids, UniProt id: Q0P8H6; denoted here as Cj1438_N_), the C-terminal truncated gene for Cj1438 (445–776 amino acids; UniProt id: Q0P8H6; denoted here as Cj1438_C_), and the gene for Cj1435 (UniProt id: Q0P8H9; denoted here as Cj1435) were chemically synthesized with codon optimization for *E. coli* expression by GenScript, USA.^27–29^ Cj1432_NMC_ was cloned into the NdeI-5′ and *Bam*HI-3′ restriction sites of the pMAL-c5X expression vector to fuse the maltose-binding protein (MBP) at the N-terminus (GenScript). A C-terminal hexa-histidine tag was also added to facilitate the purification of the protein. Cj1432_N_ and Cj1438_N_ were cloned into a pET28a expression vector with an N-terminal hexahistidine tag (TWIST Bioscience). Cj1438_C_ and Cj1435 were cloned into a pET31b expression vector with a C-terminal hexahistidine tag as reported previously.^25,30^ The amino acid sequences for Cj1432_NMC_, Cj1432_N_, Cj1438_N_, Cj1438_C_ and Cj1435 are shown in Figure S1. All proteins used in this investigation were freshly purified using the procedures described previously.^27–29^

### Synthesis of Tetramer **4**

A one pot reaction containing 20 μM Cj1432_N_, 2.0 μM Cj1432_NMC_, 20 μM Cj1438_N_, 2.0 mM UDP-Gal*f*NAc, 2.0 mM primer **3**, 2.0 mM UDP-GlcA, 2.0 mM PRPP, and 5.0 mM MgCl_2_ in 50 mM NH_4_HCO_3_, pH 8.0, was incubated for 18 h at 25 °C. The proteins were removed using a 10 K Nanosep spin filter. The reaction mixture was loaded onto a 5-mL HiTrap Q HP anion exchange column connected to an F10 NGC Chromatography System and washed thoroughly with water (10 column columns). Tetramer **4** was eluted from the column with a linear gradient of NH_4_HCO_3_ (0–50% of 500 mM NH_4_HCO_3_) and the individual fractions analyzed using ESI mass spectrometry. The fractions containing the desired product were pooled, lyophilized, and dissolved in D_2_O.

### Synthesis of Tetramer **5**

A one pot reaction containing 20 μM Cj1432_N_, 2.0 μM Cj1432_NMC_, 20 μM Cj1438_N_, 10 μM Cj1438_C_, 2.0 mM UDP-Gal*f*NAc, 2.0 mM primer **3**, 2.0 mM UDP-GlcA, 2.0 mM PRPP, 2.0 mM ethanolamine phosphate, 2.0 mM ATP, and 5.0 mM MgCl_2_ was mixed in 50 mM NH_4_HCO_3_, pH 8.0. The reaction was incubated for 18 h at 25 °C. The procedure for purifying tetramer **5** was the same as that for tetramer **4**. The fractions containing the desired product, based on mass spectrometry, were pooled, lyophilized, and dissolved in D_2_O.

### Synthesis of Tetramer **6**

Tetramer **6** was obtained by dephosphorylation of tetramer **5**. A 1.0 mL reaction containing 2.0 mM tetramer **5**, 20 μM Cj1435, and 5.0 mM MgCl_2_ was incubated in 50 mM NH_4_HCO_3_, pH 8.0, for 5 h at 25 °C. Tetramer **6** was purified using the same procedure as that for tetramers **4** and **5**. The uncharged tetramer **6** eluted in the flowthrough and individual fractions were monitored by ESI-MS. The appropriate fractions were combined, lyophilized, and dissolved in D_2_O.

### Synthesis of Heptamer **7**

A one pot reaction containing 20 μM Cj1432_N_, 2.0 μM Cj1432_NMC_, 20 μM Cj1438_N_, 2.0 mM UDP-Gal*f*NAc, 2.0 mM tetramer **6**, 2.0 mM UDP-GlcA, 2.0 mM PRPP, and 5.0 mM MgCl_2_ was conducted in 50 mM NH_4_HCO_3_, pH 8.0. The reaction was allowed to incubate for 18 h at 25 °C. The proteins were removed using a 10 K Nanosep spin filter and then heptamer **7** was purified using the same procedures as that for tetramer **4**. The fractions containing the desired product were pooled, lyophilized, and dissolved in D_2_O.

### Synthesis of Heptamer **8**

A one pot reaction containing 20 μM Cj1432_N_, 2.0 μM Cj1432_NMC_, 20 μM Cj1438_N_, 10 μM Cj1438_C_, 2.0 mM UDP-Gal*f*NAc, 2.0 mM tetramer **6**, 2.0 mM UDP-GlcA, 2.0 mM PRPP, 2.0 mM ethanolamine phosphate, 2.0 mM ATP, and 5.0 mM MgCl_2_ was conducted in 50 mM NH_4_HCO_3_, pH 8.0. The reaction was incubated for 18 h at 25 °C. The procedures for purifying heptamer **8** were the same as that for tetramer **5**. The fractions containing the desired product were pooled, lyophilized, and dissolved in D_2_O.

### Synthesis of Decamer **10**

Decamer **10** was produced in two steps. First, a reaction containing 2.0 mM heptamer **8**, 20 μM Cj1435, and 5.0 mM MgCl_2_ was incubated in 50 mM NH_4_HCO_3_, pH 8.0, for 5 h at 25 °C to produce heptamer **9**. Cj1435 was removed using a 10 K Nanosep spin filter. The filtrate containing heptamer **9** was supplemented with 20 μM Cj1432_N_, 2.0 μM Cj1432_NMC_, 20 μM Cj1438_N_, 10 μM Cj1438_C_, 2.0 mM UDP-Gal*f*NAc, 2.0 mM UDP-GlcA, 2.0 mM PRPP, 2.0 mM ethanolamine phosphate, and 2.0 mM ATP. The reaction was allowed to incubate for 18 h at 25 °C. The procedure for purifying decamer **10** was the same as that for product **5**. The fractions containing the desired product were pooled, lyophilized, and dissolved in D_2_O.

### Synthesis of Oligomeric Mixture from Tetramer **5**

Two consecutive sets of enzymatic reactions were conducted to obtain a mixture of oligomeric products. First, a reaction containing 20 μM Cj1432_N_, 2.0 μM Cj1432_NMC_, 20 μM Cj1438_N_, 10 μM Cj1438_C_, 20 μM Cj1435, 3.0 mM UDP-Gal*f*NAc, 1.0 mM tetramer **5**, 3.0 mM UDP-GlcA, 3.0 mM PRPP, 3.0 mM ethanolamine phosphate, 3.0 mM ATP, and 5.0 mM MgCl_2_ was conducted in 50 mM NH_4_HCO_3_, pH 8.0. The reaction mixture was incubated for 18 h at 25 °C and then the enzymes were removed using a 10 K Nanosep spin filter. The filtrate containing the oligomeric mixture (denoted here as **9-x**) was used to initiate a second set of reactions after the addition of 20 μM Cj1432_N_, 2.0 μM Cj1432_NMC_, 20 μM Cj1438_N_, 10 μM Cj1438_C_, 1.0 mM UDP-Gal*f*NAc, 1.0 mM UDP-GlcA, 1.0 mM PRPP, 1.0 mM ethanolamine phosphate, 1.0 mM ATP, and 5.0 mM MgCl_2_. The reaction mixture was incubated for 18 h at 25 °C and then the oligomeric product mixture (denoted here as **10–*x***) was purified using the same procedures as that described for product **5**. The fractions containing the desired products initially identified by ESI-MS, were pooled, lyophilized, and dissolved in D_2_O.

## Results and Discussion

### Synthetic Strategy

We have previously identified six enzymes required for the polymerization of the undecorated capsular polysaccharide within the HS:2 serotype of *C. jejuni* (Figure 3). To develop more robust synthesis and purification protocols for the generation of specific oligomers of defined length, we first required the identification of a suitable primer that could be used for the attachment of the subsequent sugars to the growing polysaccharide chain. We also needed a suitable purification tag that could be exploited for the convenient isolation of highly pure samples that would be suitable for ESI mass spectrometry and ^1^H NMR characterization. Toward this end we chose to initiate the polymerization reactions with the methyl glycoside of d-glucuronic acid that had been amidated with ethanolamine (primer **3**). We chose ethanolamine for the amide substituent since we could use, in subsequent steps, ethanolamine phosphate rather than the chiral (*S*)-serinol phosphate. If we initiated our reactions with one equivalent of primer **3** and one equivalent of the three activated sugars (UDP-Glc, UDP-Gal*f*NAc, PRPP) plus ATP and ethanolamine-P, we would generate a tetrameric oligomer after adding the required enzymes for the polymerization except for the phosphatase Cj1435. In such cases the polymerization process would stop since the enzyme required for the dephosphorylation was missing. The anionic nature of the phosphorylated tetramer (tetramer **5**) would enable the facile purification via anion exchange chromatography. Subsequent rounds of polymerization with the phosphorylated oligomer can easily commence by first dephosphorylating the product with added Cj1435, removal of the phosphatase, followed by the addition of another equivalent of the required substrates. The reaction sequence will always cease at the phosphorylated amide step. This strategy is illustrated in Figure 4.

### Enzymatic Synthesis of Tetramers **4**, **5**, **6**

Tetramer **4** was produced from a one-pot reaction mixture containing the enzymes Cj1432_N_, Cj1432_NMC_, Cj1438_N_, and one equivalent each of the four substrates UDP-Gal*f*NAc, UDP-GlcA, PRPP, and primer **3**. The reaction cycle ceased after the addition of the glucuronic acid moiety since amidation is required before the subsequent addition of Gal*f*NAc.^28^ The compound was isolated via anion exchange chromatography and structurally verified by ESI mass spectrometry (Figure S2). The experimental *m*/*z* value for the [M – H^+^]^−^ anion was determined to be 761.25, which is fully consistent with the calculated value of 761.25.

Tetramer **5** was synthesized using the same procedure used to make tetramer **4** except for the addition of Cj1438_C_, and one equivalent of ethanolamine phosphate and ATP. The reaction cycle stopped at the stage of the phosphorylated amide since the phosphatase Cj1435 was omitted from the reaction mixture. The negative ion ESI mass spectrum of the isolated compound is presented in Figure 5a (and Figure S3) with an *m*/*z* for the [M – H^+^]^−^ anion of 884.25, which is fully consistent with the predicted value for tetramer **5** (Figure 5b). Tetramer **5** was further characterized using ^1^H NMR spectroscopy (Figures 6a and S4). The resonances for the anomeric hydrogens of the Rib and Gal*f*NAc moieties appear at 4.81 and 5.31 ppm, respectively. The resonance for the anomeric hydrogen of the glucuronamide moiety at the reducing end of the oligomer appears at 4.81 ppm and that for the glucuronamide at the nonreducing end is found at 5.14 ppm. The resonances for the hydrogens of the −OCH_3_ and −NHAc groups appear at 3.37 and 1.97 ppm, respectively. The ratio of the integration for the four anomeric hydrogens and the hydrogens from the −OCH_3_ and −NHAc groups are 4:3:3 as expected for tetramer **5**.

**Figure 5:**
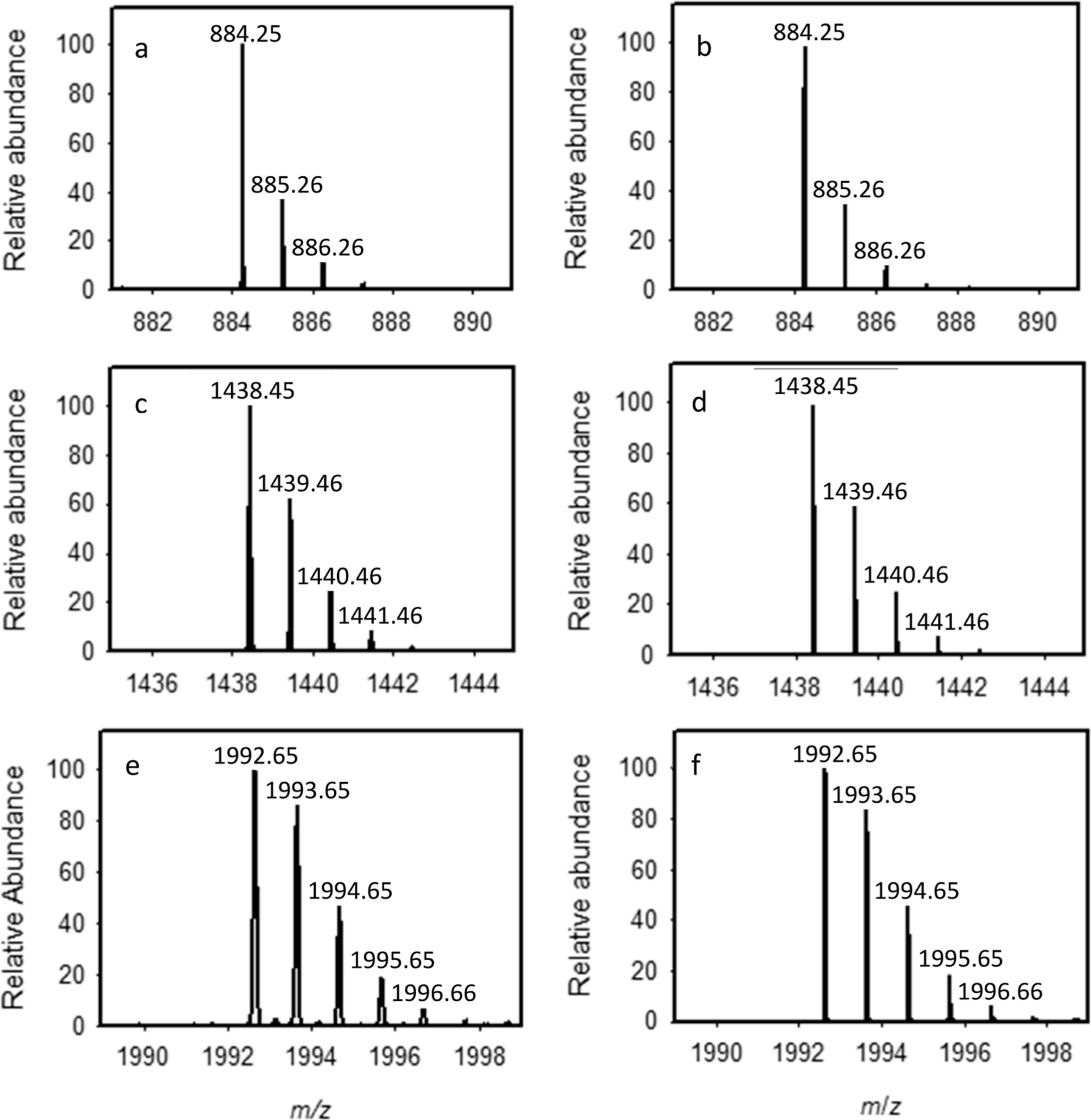
ESI mass spectral data for the [M – H^+^]^−^ anions of the isolated oligomers **5**, **8**, and **10**. (a) experimental data for oligomer **5**; (b) calculated distribution for oligomer **5**; (c) experimental data for oligomer **8**; (d) calculated distribution for oligomer **8**; (e) experimental data for oligomer **10**; (f) calculated distribution for oligomer **10**. Additional details are provided in the text.

**Figure 6:**
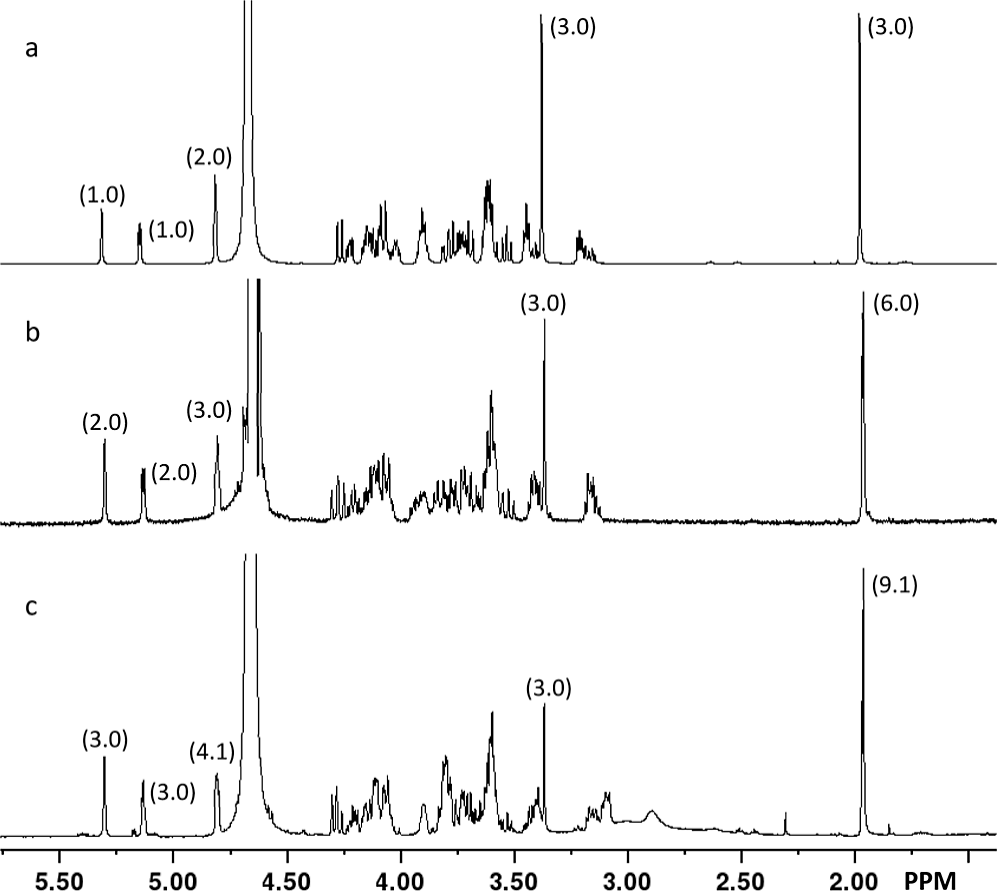
^1^H NMR spectra of isolated oligomers. (a) Oligomer **5**; (b) oligomer **8**; (c) oligomer **10**. Shown in brackets are the relative integrations for the anomeric protons between 4.75 and 5.30 ppm, the –OMe group at ∼3.4 ppm and the –*N*-acetyl group at ∼1.9 ppm. Additional details are available in the text.

Tetramer **6** was obtained by the dephosphorylation of tetramer **5** using the phosphatase Cj1435. The partially purified tetramer **6** was identified using ESI mass spectrometry (Figure S5). The observed *m*/*z* for the [M + H^+^]^+^ cation of 806.30 is fully consistent with the calculated value for this cation and is confirmed by the observation of *m*/*z* values of 828.28 and 844.26 for the [M + Na^+^]^+^ and [M + K^+^]^+^ ions, respectively.

### Enzymatic Synthesis of Heptamers **7** and **8**

The strategy for the synthesis of heptamer **7** was the same as that for the synthesis of tetramer **4** except that tetramer **6** was substituted for primer **3** (Figure 4). Heptamer **7** was purified by anion exchange chromatography and identified using ESI mass spectrometry with an *m*/*z* for the [M – H^+^]^−^ anion of 1315.44, which is fully consistent with the calculated value for heptamer **7** (Figure S6). Heptamer **8** was obtained from a one pot reaction using the same set of enzymes and substrates as for making tetramer **5**, except that primer **3** was replaced with tetramer **6**. The purified heptamer **8** was identified using ESI mass spectrometry with an observed *m*/*z* for the [M – H^+^]^−^ anion of 1438.45 (Figures 5c and S7) that is fully supported via comparison with the calculated mass spectrum presented in Figure 5d. Heptamer **8** was further characterized using ^1^H NMR spectroscopy (Figure 6b). The resonances for the 7 anomeric hydrogens appear the same as they do for tetramer **4**, except that the intensity ratios for the anomeric hydrogens, relative to those for the −OCH_3_ and −NHAc groups are now 7:3:6.

### Enzymatic Synthesis of Decamer **10**

Decamer **10** was obtained using the same strategy used for the synthesis of tetramer **5** or heptamer **8**, except that primer **3** or tetramer **6** was substituted with heptamer **8**. The purified decamer **10** was identified using ESI mass spectrometry. The negative ion ESI mass spectrum of decamer **10** is presented in Figures 5e and S8 with an *m*/*z* for the [M – H^+^]^−^ anion of 1992.65 and the calculated mass spectrum for decamer **10** is present in Figure 5f. Decamer **10** was further characterized using ^1^H NMR spectroscopy. The ^1^H NMR spectrum is shown in Figure 6c. The resonances for the anomeric hydrogens of the d-Gal*f*NAc, d-Rib and the d-GluA moieties along with –OCH_3_ and *N*-acetyl groups all appear at the same resonance positions as that for tetramer **5** and heptamer **8**, respectively, and are in the expected ratios of 10:3:9.

### Enzymatic Synthesis of Longer Oligomers

Longer oligosaccharides could potentially be made using the same strategy as outlined in Figure 4. However, a more efficient methodology is presented in Figure 7. In this case tetramer **5** is used as the initiating primer with a phosphorylated amide at the nonreducing end. This primer is mixed with the complete set of polymerizing enzymes and multiple equivalents each of the three activated sugars, including ATP and ethanolamine-P. At the end of the first incubation period a mixture of oligomeric products will be produced of different lengths whose specific composition will depend on the degree of processivity of the polymerizing enzymes. Using a ratio of three equivalents of monomers per tetramer **5**, this procedure will produce, on average, oligomers that are ∼13 monomeric units long. This mixture is depicted as oligomer **9–*x*** in Figure 7.

**Figure 7:**
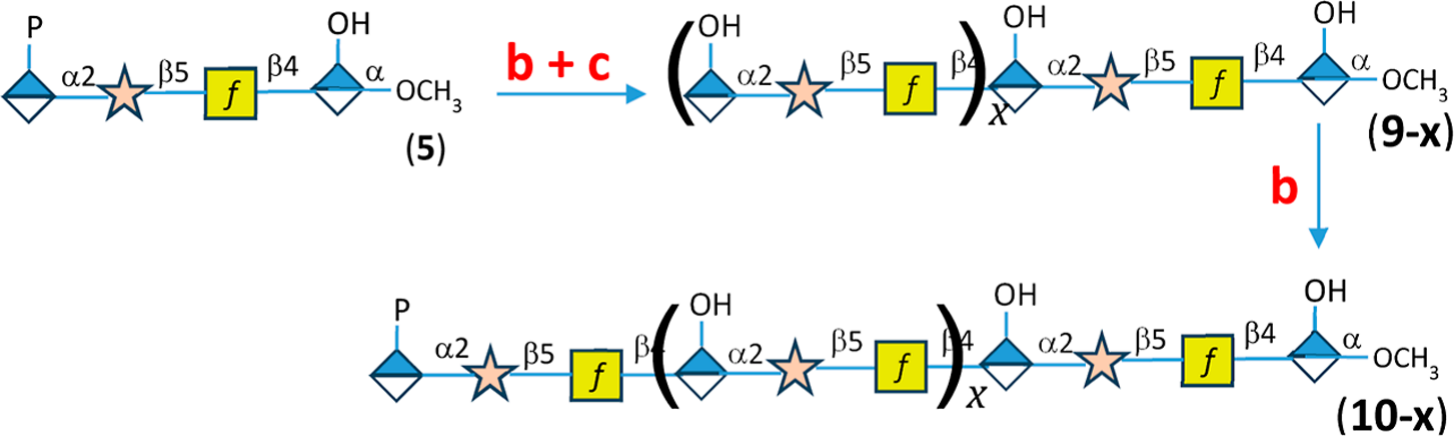
Reaction scheme for the generation of longer oligomeric products. Additional details are found in the text and in Figure 4.

To facilitate the termination of the oligomeric mixture with a phosphorylated amide, the enzymes are removed by ultrafiltration and then supplemented with one equivalent each of the three activated monomers and all of the polymerizing enzymes except for the phosphatase, Cj1435. The resulting set of oligomeric products is now depicted as **10–*x*** where the average length is expected to be 16 monomeric units. In this specific example, the mixture of oligomeric products was isolated by anion exchange chromatography as described previously. Those fractions containing the mixture of phosphorylated products were identified by ESI mass spectrometry, pooled, and lyophilized. The negative ion ESI mass spectrometry results are shown in Figures 8 and 9.

**Figure 8:**
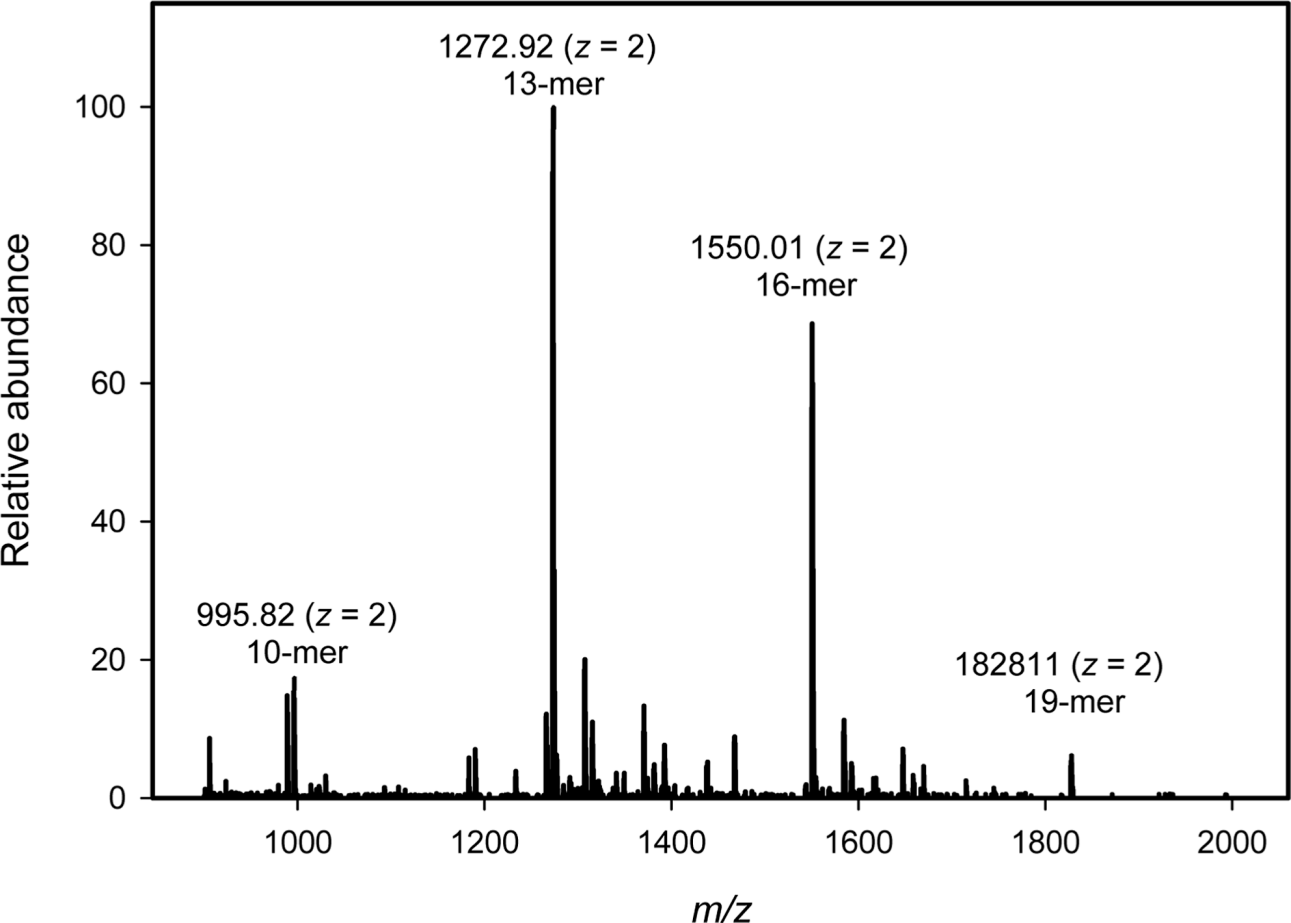
ESI mass spectrometry of the unfractionated product mixture containing compounds **10** (decamer), **10–2** (tridecamer), **10–3** (hexadecamer), and **10–4** (nonadecamer). Additional details are provided in the text.

**Figure 9:**
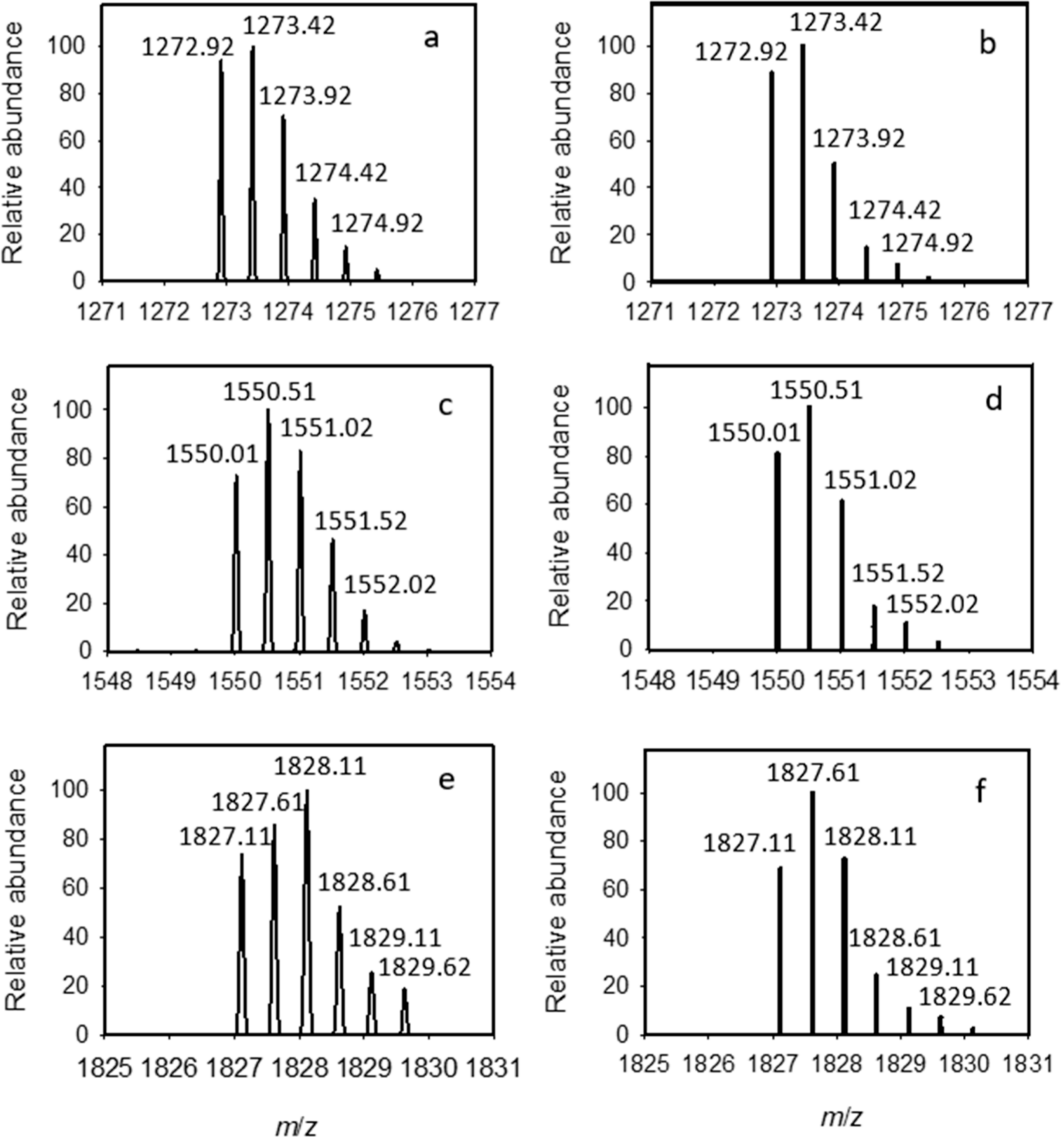
Expanded views of the isotopic clusters for the 13-mer (**10–2**), 16-mer (**10–3**), and 19-mer (**10–4**) oligomeric products identified in Figure 8. The measured and calculated mass spectra of oligomers **10–2** (a,b), **10–3** (c,d) and **10–4** (e,f).

In the ESI mass spectrum for the reaction mixture one can clearly identify the clusters of peaks that arise from the [M – 2H^+^]^2–^ dianions for the tridecamer (**10–2**), hexadecamer (**10–3**), and nonadecamer (**10–4**) via comparison of the experimental *m*/*z* values with the calculated values based on the expected molecular formula (Table 2). Unfortunately, we cannot determine the relative concentrations of the various oligomers made from the mass spectrum. The ^1^H NMR spectrum of the isolated product mixture is not sufficiently resolved to enable us to determine the ratio of the –OMe group at the reducing end of the polymer and the –NHAc groups from the GalNAc moieties within the mixture of oligomers (Figure S9). Nevertheless, we estimate that the average oligomer is ∼16 units in length.

### Enzymatic Syntheses of Other Capsular Polysaccharides

To the best of our knowledge the synthesis described here is the most complicated oligomer derived from a capsular polysaccharide that has ever been constructed starting from a simple monosaccharide primer, activated sugars, and purified enzymes. Each cycle of three monosaccharides added to the growing polymer requires six distinct enzyme-catalyzed reactions. Previously, we were able to construct the undecorated CPS from the HS:1 serotype of *C. jejuni* that consists of a repeating polymer of d-galactose and glycerol-phosphate.^31^ Oligomers of up to 17 monomeric units were detected via ESI mass spectrometry.^31^ The same d-galactose/glycerol-P polysaccharide has been synthesized using the purified polymerase (Cps7D) from *Actinobacillus pleuropneumoniae*.^32^ In another example, the capsule polymerase (CsaB) from *Neisseria meningitidis* serogroup A was used to synthesize a repeating polymer of *N*-acetyl-mannosamine-phosphate and the decorating enzyme (CsaC) and acetyl-CoA were subsequently used to add acetyl groups to the hydroxyl group at C3 of the Man-NAc moieties.^33^

## Conclusions

We have previously identified the six enzymes that are absolutely required for the polymerization of ribose, *N*-acetyl-galactosamine, and glucuronic acid during the assembly of the undecorated capsular polysaccharide from the HS:2 serotype of *C. jejuni*.^27–29^ Here we developed a robust and efficient enzymatic strategy for the synthesis of well-defined oligosaccharides of specific lengths. Our novel procedure relies on the biochemical observation that polymerization cannot continue unless a specific phosphorylated intermediate is hydrolyzed. Using this methodology, we were able to isolate and chemically characterize oligomers that were 4-, 7-, and 10-monomeric units in length. Variations of this technique allowed mixtures of oligosaccharides to be synthesized containing at least 19 monomeric units. The ability to construct oligosaccharides in this manner will now enable the elucidation of the enzymatic pathways for decoration of these polysaccharides with other sugars and methyl phosphoramidate modifications. The preparation of these oligosaccharides may further facilitate the synthesis of glycoconjugates that could serve as precursors to the development of vaccines that target specific strains of *C. jejuni*.

## Supplementary Material



## Notes

Amino acid sequences of the purified proteins, and ESI-MS and NMR spectra of enzyme-catalyzed reaction products (PDF)
