# Supplementary material for: Enzymatic Synthesis of an Undecorated Capsular Polysaccharide from Campylobacter jejuni
Source: Biochemistry. 2025 Aug 11;64(17):3771–80. doi: 10.1021/acs.biochem.5c00314 (PMC12409886; doi:10.1021/acs.biochem.5c00314)
Supplement: Supplementary file 1 [file bi5c00314_si_001.pdf]

Supplementary Information

Enzymatic Synthesis of an Undecorated Capsular Polysaccharide from *Campylobacter jejuni*

Dao Feng Xiang, Tamari Narindoshvili, and Frank M. Raushel*

Department of Chemistry, Texas A&M University, College Station, TX 77843, United States

*Contact Information

e-mail: raushel@tamu.edu

(a)

MKIEEGKLVIWINGDKGYNGLAEVGKKFEKDTGIKVTVEHPDKLEEKFPQVAATGDGPDIIFWAHDRFGGYAQSGLL
AEITPDKAFQDKLYPFTWDAVRYNGKLIAYPIAVEALSLIYNKDLLPNPPKTWEEIPALDKELKAKGKSALMFNLQE
PYFTWPLIAADGGYAFKYENGKYDIKDVGVDNAGAKAGLTFLVDLIKNKHMNADTDYSIAEAAFNKGETAMTINGPW
AWSNIDTSKVNYGVTVLPTFKGQPSKPFVGVLSAGINAASPNKELAKEFLENYLLTDEGLEAVNKDKPLGAVALKSY
EEELVKDPRIAATMENAQKGEIMPNIPQMSAFWYAVRTAVINAASGRQTVDEALKDAQTNSSSNNNNNNNNNNLGIE
GRISHMIKKILLITPELEYTGALNSFKRICEVLLNNKYAVDIWTYNEGPYISEFDKLGVYYVEVISEDDIDSKWVHER
ISKYSLVIANTIVVYKCVELIQNLTPVVWYIREAENLPDFFWKPERKLALEKAKKLYVVSEYAKDFIIHNYNKNVEV
LHNYVDDVFYEKHDDFLKQIKSDKLKFLALGTIEKRKGYDVLLQAFIDLPVDIRDQCELHFAGRFWEGAKDFFPKIL
SLAKKFPNIFYHGELRDRKKIHSLIFQCNVMVVPSRDESCSLVALEGAMMSKPLILTENIGAKYILDENSGWLVKTG
SVDSLKNAFIQAYKNKNKLDAMGANSRNNYLQTSTYEIYEKNILKMVRDEICKNQYLYRINQENYVLFSFDIFDTLI
SRNIAKPSAVFLIMKQKMRNMDFPLNLVKNFDRIRVEVEQYYYRNVCKNKYEDTNFDEIYNLLQQNFSLSFQQKEEL
MKLEINTEKETLYPIKKNIELVEELIKNEKRVVLISDMYFSSSIIRTFLNKFSPIFNNIPIYMSSEFRLKKNSGNLF
KAILNLEKVDPKKWIHCGDNWVGDYLKPSNLEISTNFYINQLLPYEEFALNRNSLDMDLQKIIGISKKIRLENTLTN
LQEIGVSFGAPMLLPYVQWILNIALKNSIRCLYFIARDGYVLQKMTDMLIQAKKINIKTKYLYGSRESWREPFRNKD
KLKIQLIDEYLDQEIDKQEIFAFVECCGTGETLDYIVKRIESNQQFKNMFFGSLYLYRSKLNKTKTQSLFMLPLNEN
YTYGIELFVRSLQGQVLGYDKKDGRVIPVFDFLEGEALQKFRYDEYIDGVMLFMEYIVKTDNYEKIFDNMNVTILYL
NYLSNNYIDKKFIEIMGNVPFILNGVKDRVGIFAPRLNNKITLDQKNSFFNWSVLRSCKDIRVKYNMDNDCYSSENL
YFQGSSHHHHHH

(b)

MGSSHHHHHHSSENLYFQGHMIKKILLITPELEYTGALNSFKRICEVLLNNKYAVDIWTYNEGPYISEFDKLGVYVE
VISEDDIDSKWVHERISKYSLVIANTIVVYKCVELIQNLTPVVWYIREAENLPDFFWKPERKLALEKAKKLYVVSEY
AKDFIIHNYNKNVEVLHNYVDDVFYEKHDDFLKQIKSDKLKFLALGTIEKRKGYDVLLQAFIDLPVDIRDQCELHFA
GRFWEGAKDFFPKILSLAKKFPNIFYHGELRDRKKIHSLIFQCNVMVVPSRDESCSLVALEGAMMSKPLILTENIGA
KYILDENSGWLVKTGSVDSLKNAFIQAYKNKNKLDAMGANSRNNYLQTSTYEIYEKNILKMVRDEICK

(c)

MGSSHHHHHHSSGLVPRGSHMMNYNTPKVSIVVPSLNSISYIRECIDSILNQTLKDIEILCIDANSTDGTLEVLKNY
EKKDKRLRVIISDKKSYGYQMNLGIKEAKGEYLGIVESDDYIKTNMYERLYEIAKKNDCEVVKGDFYILESNKGKYS
KITPIDFLYNQIISFKTHPNIFNFQSINPIGIYRLDLLRTNQIKLNETPGASYQDNGLWFQIFALAKSIYFINEAFY
MLRRDNPNSSVKSKEKVYCACEEYDFIRDFLKKHPDLEKTLAPICALHRFGNYMFTLERIDERYKLDFLKRFSQDFR
KILKDKELDENLFGDGDMKIIYSIVENPENYYFLYMG

(d)

MKELSDYDFLLNRHKQIFDYTPDFKCPVTFNEKLIYRILYDRSCIYSFLADKIKMRFYVASALSDNHEYSWDKIDIL
NEKSILFNNIDDLQDKIFETNKCKYLPKIYGIYKNIYDINFNELPNSFVLKTNHDCGGYVIVENKQEFLRDTVVFSN
AMKKLKKHLEWNYYSVFREWHYKDIEPRVFAEELLLGENKKPADTYKFHIFDKENLSNNFIQVTTDRFDNYQRAMFD
LSWNLAPFNFMYDNKNVTMIPKKPNLLDSMINISLILAKPFDYVRVDLYQFDKKIYIGELTFTHGAAGEKVIPKEWD
KKLGDLWRLKRLDNASKLEHHHHHH

(e)

MSKEILALFDFCETLTNFQTLDRYLPLAGSKNINYTQSKNLARRERFQRENLPYPRYEWLIDLDVDLAEEIAQEFVY
TDVMANLNQNVMDRLFWHQDEGHTIVIVSGGLTIYIKEFARIYNIENIVAVDLEIYKNKLTGNIDGIHTMQERKLYK
LAQKFNLKQFDLKNSYAYSDCVSDIPLLSLVGNPNVIECGKDLQWARILGFNILLKYLEHHHHHH

Figure S1: The amino acid sequences for the 5 proteins used in this investigation. (a) MBP-Cj1432$_{1-914}$ (denoted as Cj1432$_{NMC}$). The sequence of the maltose binding protein (MBP) tag is colored green. The sequence of Cj1432$_{1-914}$ is colored black, and the linker between the MBP and Cj1432$_{1-914}$ is colored purple. The polyhistidine tag at the C-terminal end is shown in red. (b) Amino acid sequence for Cj1432$_{N}$. The polyhistidine tag at the N-terminal end is shown in red font. (c) Amino acid sequence for Cj1438$_{N}$. The added polyhistidine purification tag at the N-terminal end is shown in red font. (d) Amino acid sequence for Cj1438$_{C}$. The added polyhistidine purification tag at the C-terminal end is shown in red font. (e) The amino acid sequence for Cj1435. The added polyhistidine purification tag at the C-terminal end is shown in red font.

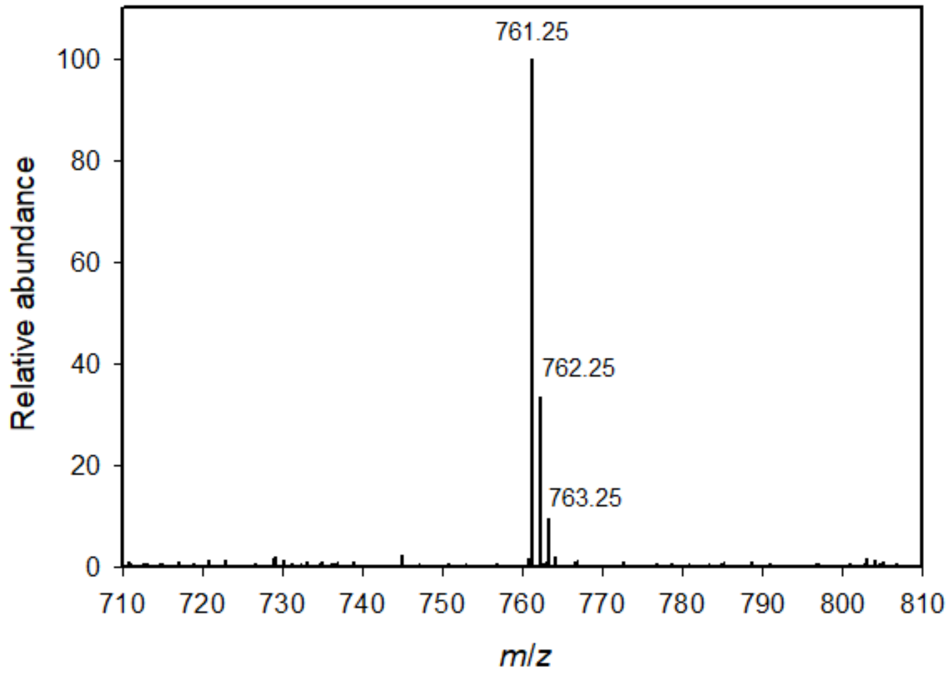

Figure S2: ESI-MS results for the $[M–H^{+}]^{-}$ anion of tetramer **4**.

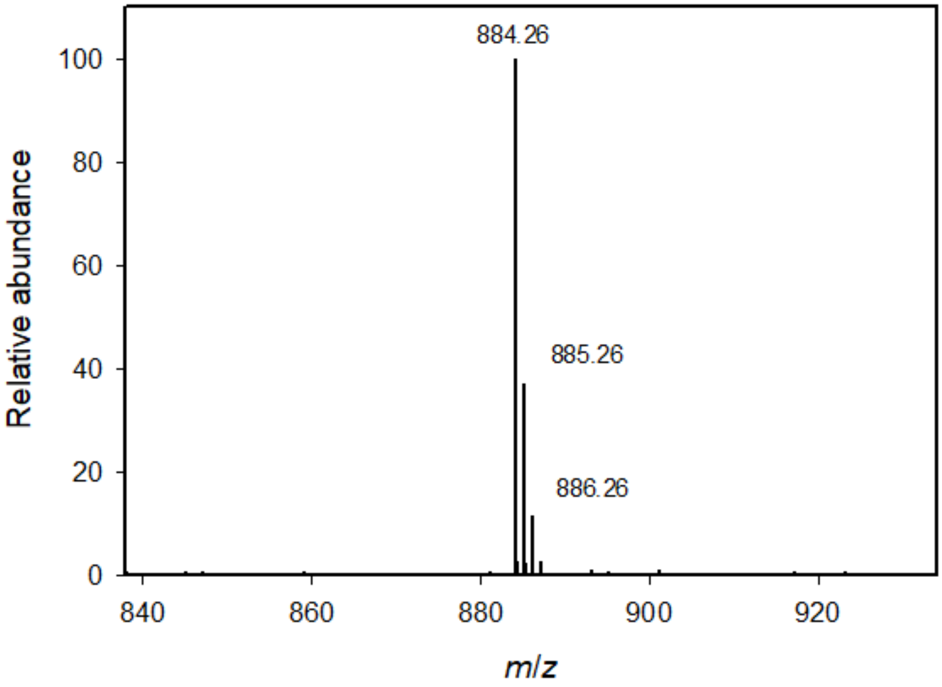

Figure S3: ESI-MS results for the [M–H$^+$]$^-$ anion of tetramer **5**

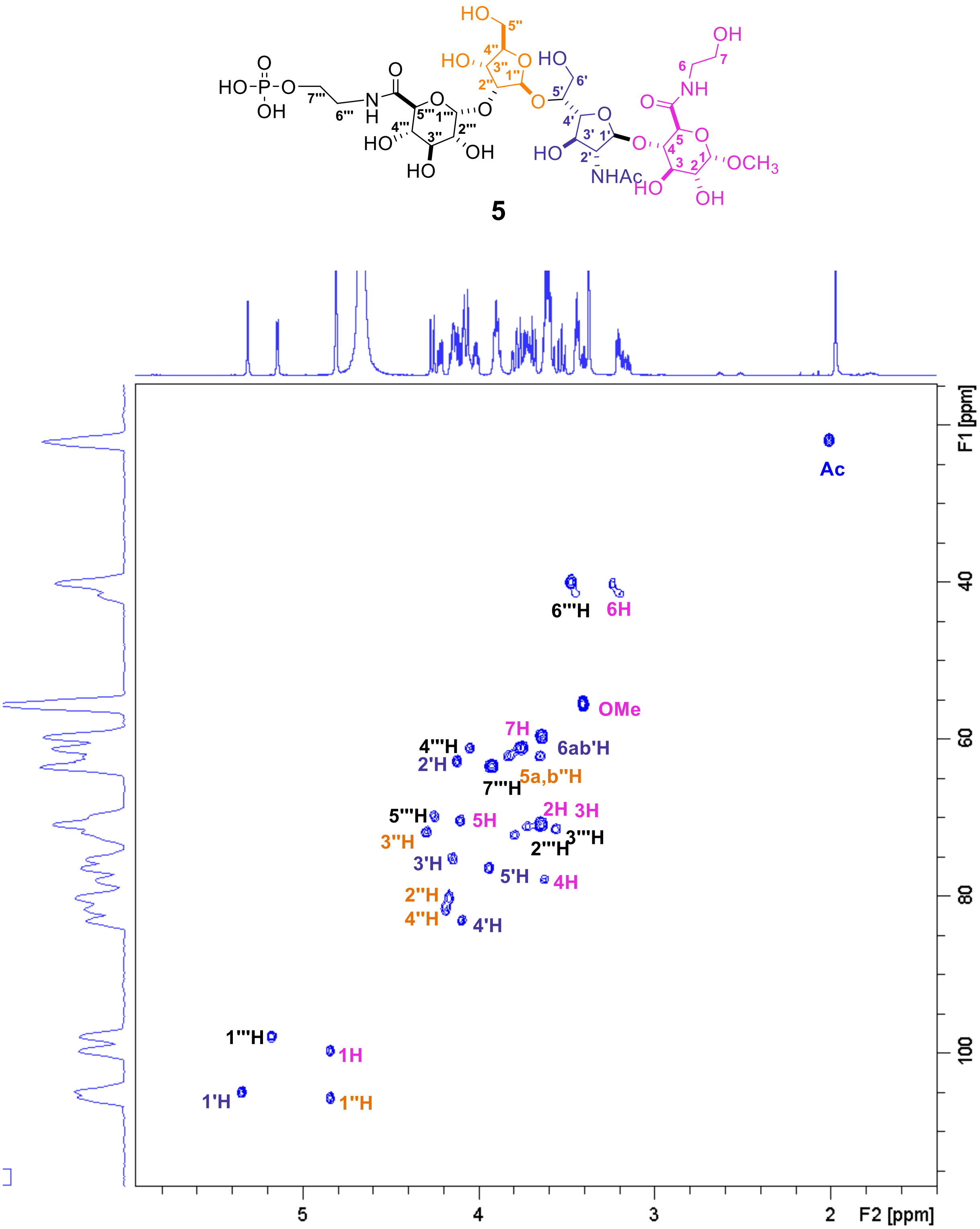

Figure S4: HSQC NMR spectrum of tetramer **5**.

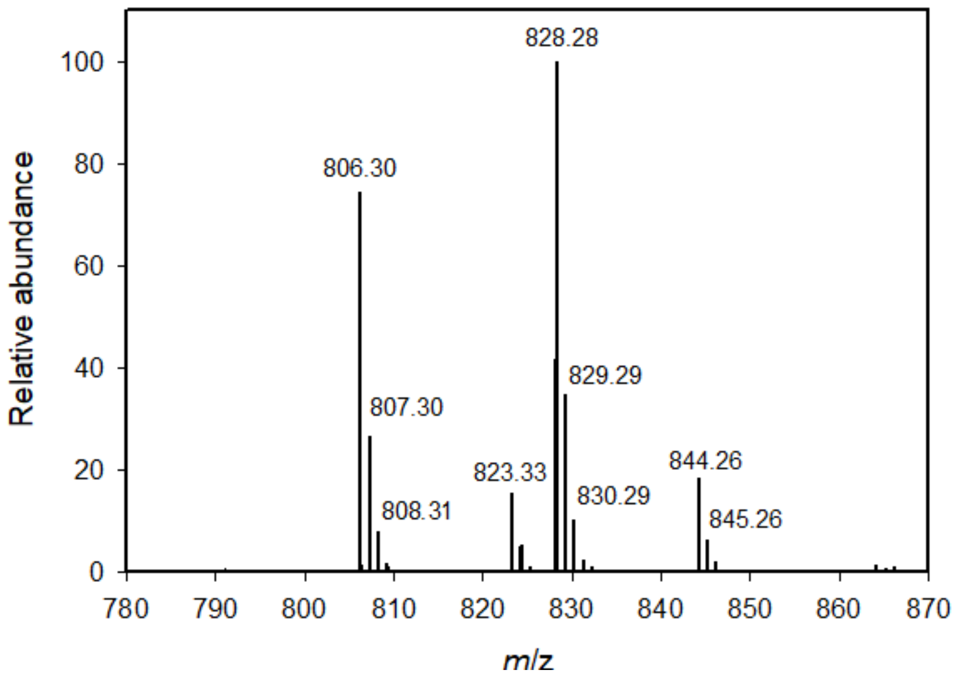

Figure S5: ESI-MS results showing the $[M + H^+]^+$, $[M + Na^+]^+$, and $[M + K^+]^+$ cations for tetramer **6.**

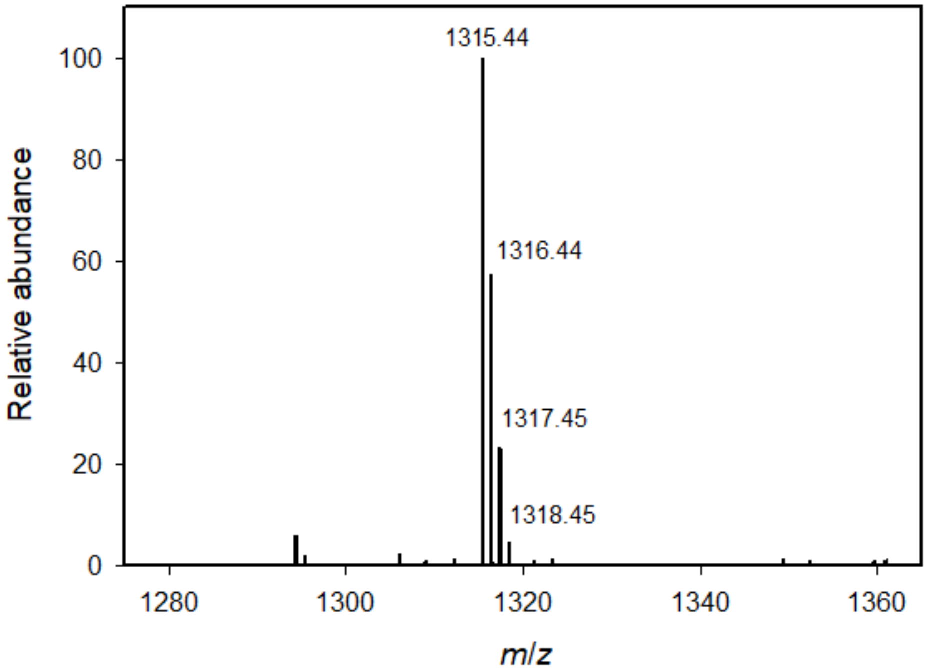

Figure S6: ESI-MS results for the $[M{-}H^{+}]^{-}$ anion of heptamer **7.**

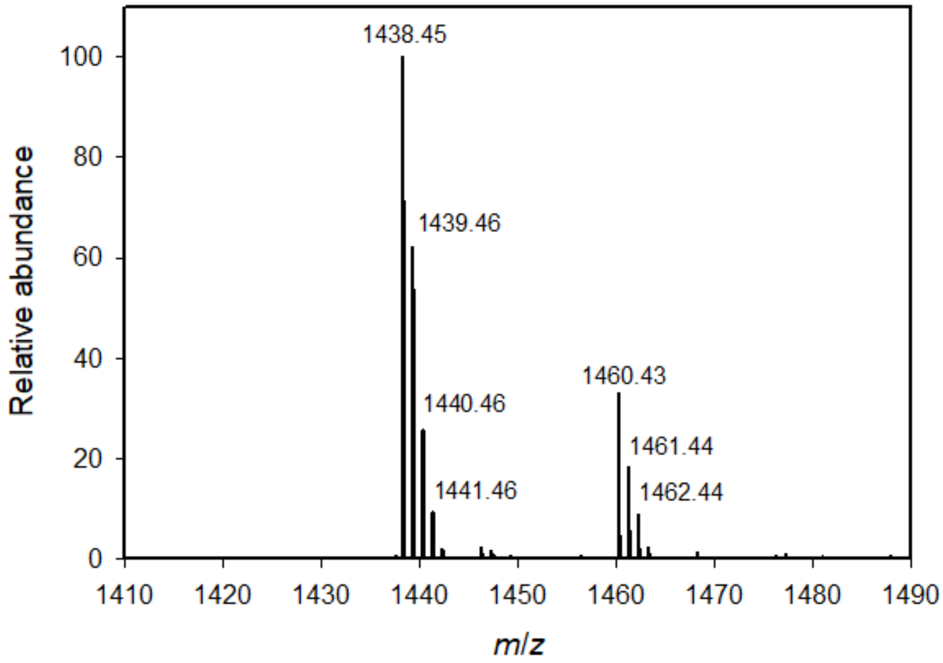

Figure S7: ESI-MS results for the $[M{-}H^+]^-$ anion of heptamer **8.** The *m/z* of 1460.43 is consistent with the $[M - 2H^+ + Na^+]^-$ anion of heptamer **8.**

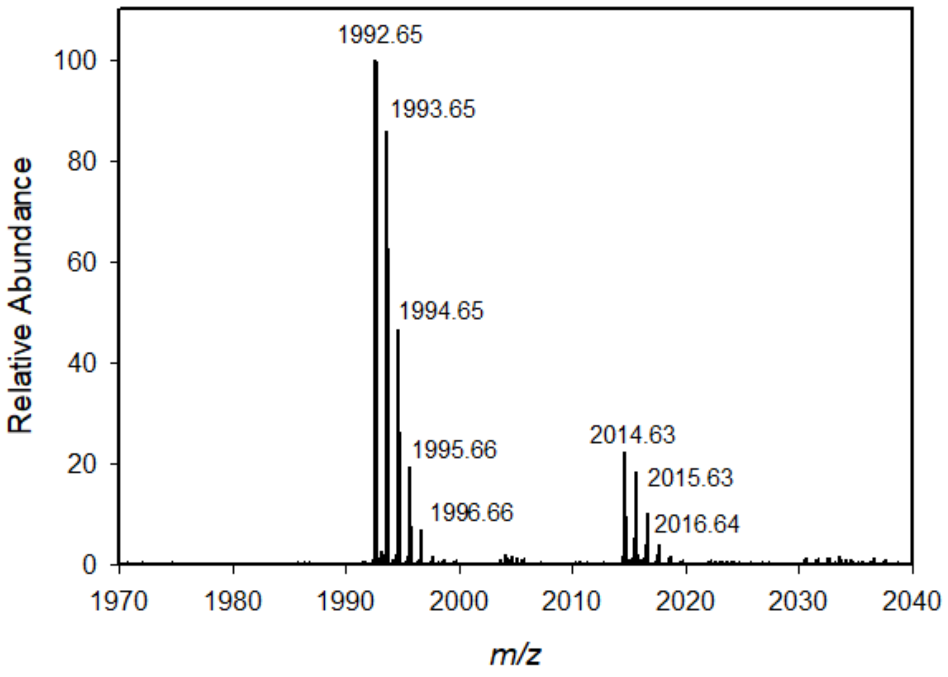

Figure S8: ESI-MS results for the $[M{-}H^+]^-$ anion of decamer **10**. The *m/z* of 2014.63 represents the $[M - 2H^+ + Na^+]^-$ anion of decamer 1**0.**

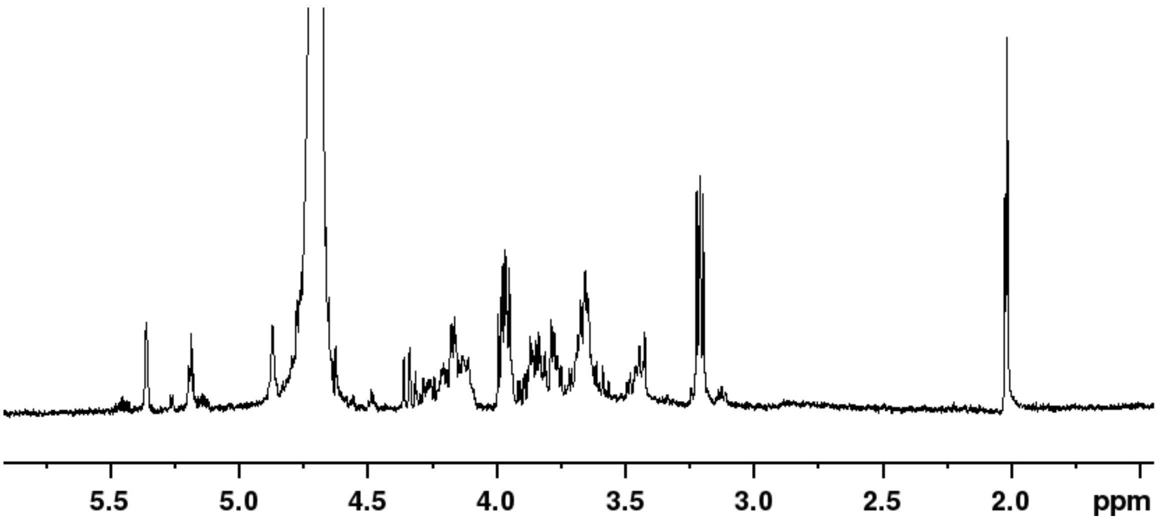

Figure S9: ^{1}H NMR spectrum for the oligomeric mixture (**10-x**). Additional details are provided in the text.
